# Evolutionary Thinking in Microeconomic Models: Prestige Bias and Market Bubbles

**DOI:** 10.1371/journal.pone.0059805

**Published:** 2013-03-27

**Authors:** Adrian Viliami Bell

**Affiliations:** Department of Anthropology, University of Utah, Salt Lake City, Utah, United States of America; Cinvestav-Merida, Mexico

## Abstract

Evolutionary models broadly support a number of social learning strategies likely important in economic behavior. Using a simple model of price dynamics, I show how prestige bias, or copying of famed (and likely successful) individuals, influences price equilibria and investor disposition in a way that exacerbates or creates market bubbles. I discuss how integrating the social learning and demographic forces important in cultural evolution with economic models provides a fruitful line of inquiry into real-world behavior.

## Introduction

Cultural variation is widespread and important to economies [1]. Cultural-prescribed behavioral diversity in domains such as commons institutions [2], languages [3], and the formation of ethnic groups [4] provide challenges and opportunities to economic development and governance. Interactions across linguistic or ethnic groups influence fairness preferences [5], labor wage differentials, high communication costs may lead to bargaining breakdowns [6], [7], and diverse ethnic groups may undermine political bargaining leading to gridlock [8]. On the other hand, diverse communities may also promote production and innovation by the more frequent introduction of novel ideas [9], [10]. Behavioral scientists must account for the origin of cultural diversity and change before arriving at an understanding important for policy makers and theoreticians alike.

In this paper I discuss the gene-culture coevolutionary approach to explaining cultural variation and briefly introduce its theories of individual decision making. Since much of the theory is about the evolution of learning, it offers a simultaneous appeal to the ultimate origins of behavior as well as the proximate mechanisms behind behavioral change as actors adopt (or not) behaviors from others. After briefly reviewing the theoretical and empirical evidence for social learning biases, I show how actors engaging in a prestige bias, in particular, alter price equilibria using a stylized model of price dynamics.

### Gene-culture Coevolution

Among primates, humans are an anomaly for their ability to generate highly variable speech. Key to our ability is the much lower position of the larynx, or “voice box”, allowing us to easily produce a far more diverse repertoire of discriminable vocal tract resonances compared to other primates [11]. While there are a fair number of hypothesis for how this first arose [13], it is highly plausible that the human vocal tract has been modified and maintained to enhance the capacity to acquire and use cultural information. In a like manner that human morphology was shaped by social influences, especially regarding cultural learning, selection is likely to have played a role in honing our ability to acquire cultural information in adaptive ways [13].

**Figure 1:**
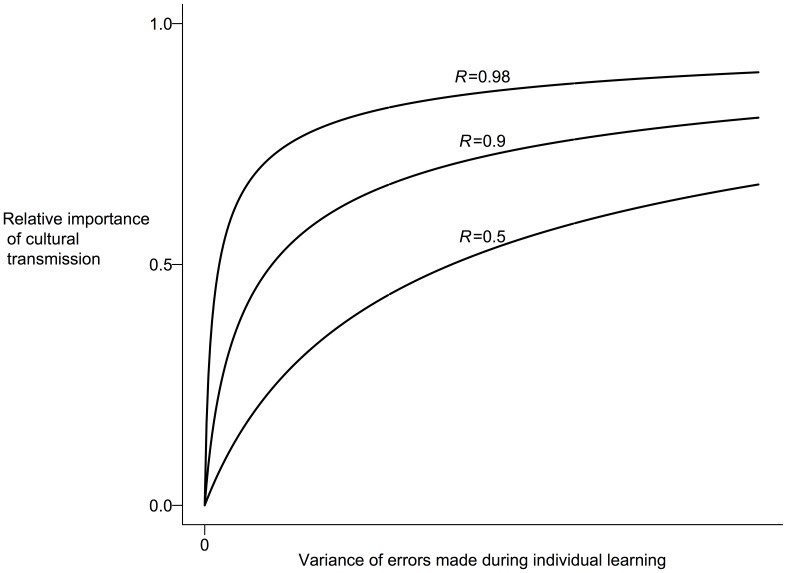
When culture (tradition) is favored in a fluctuating environment. A result of an evolutionary model of learning ([13], chap. 4), plotted are three curves for three values of environmental autocorrelation (*R*). Higher values of *R* indicate more similar environments through time. This result extends to Bayesian learners and environmental heterogeneity [17]. Social learning (e.g. imitation or adherence to tradition) is favored by natural selection and will maximize utility as environments change less and individual learning becomes more error prone.

The human capacity for complex culture likely began 250,000 years ago [14], and since then has generated adaptive culture that allowed human populations to expand to the most marginal regions of the world. Human populations have long faced a widely changing environment both spatially and temporally, and theoretical models show how natural selection would favor individuals to engage in optimal amounts of adaptive social learning and individual innovation ([13], chap. 4). For example, the turbulent Pleistocene environment presented the right conditions to favored the evolution of savy social learners ([15], pp 131–139), and the relative stability of the Holocene may have favored accumulated cultural knowledge in plant innovations leading toward domestication [16]. In fact, adherence to tradition can maximize utility better that Bayesian learning under certain conditions [17]. In a fluctuating environment, adaptive learners pay more attention to tradition as the environmental autocorrelation increases and the quality of environmental information decreases (Figure 1).

The consensus among many is that humans have an evolved psychology to learn. Evolutionary theoreticians [13], [15], [18] and economists [19] together have motivated a number of social learning biases that, scaled to the population level, can lead to distinct cultural trajectories. Behind this theoretical foundation, a growing field of experimental and field studies are bringing us closer to a finer description of how actors adopt behaviors and beliefs.

### Evidence for Learning Biases

While the evidence clearly suggests our preference to imitate others is extraordinary among primates [20], there is a slow developing consensus on how and when humans learn socially. Experimental evidence in anthropology and psychology show that individuals have a propensity to imitate the successful in economic games [21] and conform to majority behavior in other experimental tasks [22]–[24]. In a multi-armed bandit, McElreath et al. [21] show participants preferring to imitate those with higher payoffs. It is likely that actors employ a number of social learning strategies, with substantial heterogeneity within a group [24]. Ethnographic accounts suggest individual biases in cultural learning are behind the persistent ethnic subcultures that maintain substantial differences in norms of violence, farming practices, and religious beliefs [25], [26].

These findings suggest a subset of learning strategies may be especially important to microeconomic processes (Table 1). One particularly salient bias is the copying of prestigious individuals, a Model-based bias, where actors adopt the behaviors or beliefs from those who receive a disproportionate amount of attention or deference from other learners (i.e. Warren Buffet). Perhaps a universal feature, human societies recognize the prestigious who are liable to be imitated by many. Evolutionary models suggest prestige biased learning as a broadly adaptive learning strategy as it provides a quick way for a naive learner to obtain optimal behaviors in the current environment (see [27] for a review of the theory and evidence). In an attempt for direct experimental evidence for a prestige bias, [28] show that in certain conditions children preferentially learn from those who receive more bystander attention. Even among chimpanzees with relatively limited social learning abilities compared to humans, there is evidence of preferentially adopting solutions to a foraging problem from higher-ranked and older chimpanzees [29]. Clearly, this social learning “force” should be incorporated into our models of decision making.

**Table 1:** Plausible learning biases important to economic processes – decision-making and biased-transmission learning strategies (from [15]).

Decision-making forces
**Guided variation.** Nonrandom changes in cultural variants by individuals that are subsequently transmitted. This force results from transformations during social learning, or the learning, invention, or adaptive modification of cultural variants.
**Biased-transmission**
**Content-based (or direct) bias.** Individuals are more likely to learn or remember some cultural variants based on their content. Content-based bias can result from calculation of costs and benefits associated with alternative variants, or because the structure of cognition makes some variants easier to learn or remember.
**Frequency-based bias.** The use of the commonness or rarity of a cultural variant as a basis for choice. For example, the most advantageous variant is often likely to be the commonest. If so, a conformity bias is an easy way to acquire the correct variant.
**Model-based bias.** Choice of trait based on the observable attributes of the individuals who exhibit the trait. Plausible model-based biases include a predisposition to imitate successful or prestigious individuals, and a predisposition to imitate individuals similar to oneself.

To motivate the use of social learning forces in economics, I use a model to illustrate how biases in social learning can influence price dynamics in a market. First I consider a model of herding behavior by Lux [30], and show how biases in social learning can influence price equilibria. Then I show how a prestige bias can cause or exacerbate market bubbles. As a simple caricature of a complex problem, the goal is to highlight in a transparent fashion the effects of an evolutionary-motivated learning strategy on asset price and investor disposition equilibria.

## Analysis

Since prestige bias was likely a good learning strategy for much of our species history, lets assume that actors have a culturally and/or genetically reinforced propensity to learn from prestigious persons. Given this propensity, the goal is to see how trade price equilibria may significantly differ from their fundamental value as investors learn, then buy or sell.

Investing in inflated markets through herding behavior has been modeled previously by Lux [30], and I will use this modeling framework to describe the effect of theoretically motived social learning strategies on market price equilibrium. Let 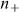 and 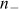 be the number of optimistic and pessimistic investors, respectively. By defining 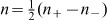, 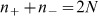, and 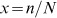, then *x* is an index from −1 to 1 describing the average disposition of investors. Increasing lower and negative values indicate more pessimistic investors (sellers), increasing higher and positive values indicate more optimistic investors (buyers), and 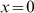 indicates an equal number of buyers and sellers. Let 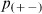 and 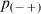 be the transition probability of investors who are pessimistic becoming optimistic, and those who are optimistic becoming pessimistic, respectively. Then a recursion describing the disposition of investors is:
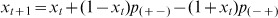
(1)


For a model of price dynamics, following [30], assume that pessimistic investors enter the supply side by selling the asset and optimistic investors enter the demand side by buying additional units of the asset. Each trader buys or sells a fixed amount of stock (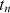), and the total trading volume of speculative investors becomes 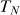. Then the net excess demand becomes:
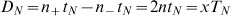
(2)where 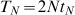, the total trading volume for this group of speculators. At this point, for all trades to be completed it is required that 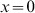. Therefore a second group of traders is added that would buy or sell to speculators. Call these traders fundamentalists, such that excess demand of this group depends on the difference in the fundamental value of the asset (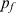) and the actual price at time 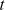, (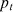).
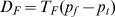
(3)where 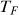 is the total trading volume of this group. Assuming that prices respond to excess demand in supply, translated in price change by constant 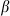, we can then derive recursions describing the price dynamics.



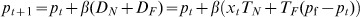
(4)The full system of investor behavior and price dynamics then becomes:
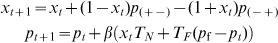
(5)


Our analysis focuses on how how learning can affect price equilibria (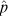) from their fundamental price (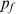), and the equilibrium disposition of investors (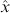). The price equilibria,
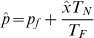
(6)depends on the final disposition of investors. The key learning process occurs in defining the transition probabilities from buyer to seller and vice versa (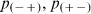). If we let the transition probabilities equal the current frequency of an investor strategy, i.e.,
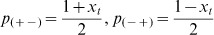
(7)then it is easy to see that the system (5) yields no change in x (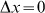). This fundamental result in cultural evolution suggests that cultural change occurs when there are biases in social learning, such as actors assorting or learning preferentially from those with similar preferences [13].

Instead of adopting the disposition of random individuals, assume that actors follow current market trends such that dispositions change depending on accurate projected directions of the trade price. An increase in the market value will more likely cause pessimistic speculators to become optimistic (buyers), and a decrease in market value will cause optimistic investors to more likely become pessimistic (sellers). Then the transition probabilities become:
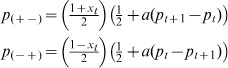
(8)


The equilibrium, (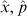), following these transition probabilities is (0, 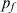). The eigenvalues of the linearized system (5) shows that this equilibrium (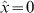, 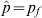) is stable when one of the below two conditions are satisfied (see Supporting Information, Derivations S1):
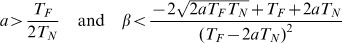
(9)or if,



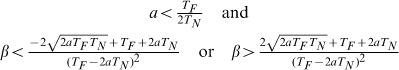
(10)Remembering that 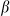 translates the excess demand into a change in trade price and parameter 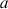 scales the effect of price projections on investor disposition, the above condition reflects the level of response to demand and price changes. This makes sense since [30] shows (in the continuous time version) that an unstable equilibrium at (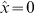, 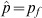) leads to limit cycles, and cycles generally depend on time lags – in this case responses to price and demand changes. Even when additional “mood of the market” parameters heighten the influence of social contagion that would potentially dampen the cycles, the cycles persist because price increases diminish and the market “loses confidence” [30]. Neither the state of pure optimism (or pessimism) can exist indefinitely [31].

Finally, consider a prestige bias such that individuals adopt the behavior of successful individuals. Rather than follow market trends, actors may look toward prestigious individuals whose assets are higher valued. An indirect measure of this success may be how different the fundamental value of an asset is from the current price of the asset (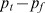). The individuals with the high-valued asset, relative to the fundamental price, are viewed as opinion leaders that prescribe the pathway to success. When actors meet, they learn of others’ updated assets and assume the net increase in asset value to be the difference from the fundamental price. Actors compare this difference to a baseline difference, (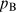), that may represent a shared preference or some outside option. Given that an actor meets an individual with an opposite disposition, she may adopt a disposition depending on this difference scaled by a constant (*a*). The transition probabilities become:
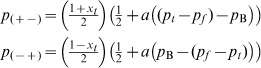
(11)


This specification leads to the investor-price joint equilibria (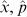) as (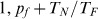), (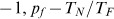), and (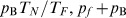 ). Equilibrium trade price in the state of pure optimism (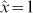, the “growing bubble” condition) depends on trade volume between our two groups and the fundamental price:
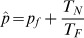
(12)


This equilibrium has the potential to increase indefinitely as the fraction of trading originates from non-fundamentalist speculators. Under prestige-biased learning specified in (11), the equilibrium with only optimistic investors is stable when 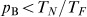 (see Supporting Information, Derivations S1). The greater the relative trading volume for non-fundamentalist than fundamentalist investors, the greater the chance for most investors feeling optimistic about their assets in the long run. In other words, the more traders using prestige biased social learning the higher and more stable are inflated assets. This outcome is limited when assets in outside markets signal increasing high returns (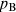) from their own fundamental price. Note also that the pessimistic equilibrium is always stable and the unstable internal equilibrium, (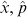) = (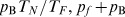 ), which determines the domain of attraction for the pessimistic and optimistic equilibrium, becomes increasing in favor of pessimism with a *larger* fraction of the trading coming from non-fundamentalists. In sum, if outside markets present minor “prestigious” gains relative to the current market and there is a precedence for optimism among investors, then prestige biased decision making among speculators can prolong an “irrational” state of optimism and lead to or exacerbate market bubbles.

## Results and Discussion

This model shows how an evolutionarily favored social learning strategy can cause an “irrational” inflated price equilibrium. Investors able to forecast market trends will keep price dynamics in the region of the fundamental price (perhaps cycling around it), thus controlling price inflation. Once actors imitate the disposition of prestigious individuals, along with control of a disproportionate share of the trading volume, prices inflate leading to a potential market collapse.

Other explanations for market bubbles may fit into the intuitions of this model. One narrative argues that herding among traders is due to reputational effects and the relative performance of mutual fund managers. Dass et al. [32] found that managers that were payed on an absolute scale documented less herding effects than among fund managers evaluated on a relative scale with other managers. Along the same theme, DeMarzo et al. [33] use a model to argue that rational actors with relative wealth concerns may not trade against the crowd. This result is driven by the fact that they include the wealth of others in their utility function.

This model analysis further corroborates thinking on economic contagion that posits a number of mechanisms by which shocks propagate more than expected [34], one of which emphasizes the role of incomplete and/or asymmetric information ([35], e.g.). Here social information is predictive as investors follow “informed” market participants, subjecting the market to fads and rumors leading to herding behavior. As shown above, using social influence is broadly favored by natural selection when the cost of accurate information is high (Figure 1). Therefore, one or more of the social learning biases in Table 1 may characterize how investors behave.

Future work is needed to directly test empirically whether investors adopt the behaviors of prestigious individuals and therefore exacerbate market bubbles. An effective assessment would include broader theoretical development than presented here, as the above stylized model of markets is to motivate future theoretical work as well as empirical inquiry (i.e. when the fundamental price is difficulty to assess, as in [36]). Also, since it is likely that humans may use a variety of different social learning strategies ([24], e.g.) that may mitigate or further exacerbate market bubbles, models assuming heterogeneity in learning strategies would likely yield new insights and predictions for empirical testing. Further, proponents of the rational actor concept may derive conditions under which prestige bias, or any other evolutionary-motivated social learning strategy, yields rational decision-making.

### Integrating Other Evolutionary Insights

Gene-cultural coevolution motivates social contagion behaviors often invoked in economic models. However, applying evolutionary theory may also move beyond the social learning preferences actors use, and consider other important parameters in gene-culture coevolutionary research. Population size, migration rates, and other demographic parameters have shown in theory to affect cultural variation. Especially in small groups, technology may be lost due to random loss of experts, especially for harder to learn objects ([13] chap. 7, [37]). Engineers may “disinvent” innovations, and a weak link in the chain of knowledge reproduction may result in the loss of innovations. Heavy migration may increase the effective size of the cultural traits available to learners, and may make populations more similar, depending on the learning strategies employed, migration patterns, and the strength of local adaptation [38].

### Conclusion

This paper demonstrates the scope of the gene-culture coevolutionary approach to explaining economic behavior in markets. Through a simultaneous appeal to proximate mechanisms and ultimate origins, an analysis of market behavior provided novel results, suggesting further theoretical and empirical work on social learning strategies. Investors able to forecast market trends will keep price dynamics in the region of the fundamental price (perhaps cycling around it), thus controlling price inflation. However, once actors imitate the disposition of prestigious individuals, along with control of a disproportionate share of the trading volume, prices inflate leading to a potential market collapse. By integrating social learning strategies and other evolutionary forces into economic models, new avenues of inquiry are likely to open and provide new insights into complex economic phenomenon.

## Supporting Information

Derivations S1 **This technical appendix reports the equilibria and stability conditions discussed in the main text.** Further details can be requested from the author.
(PDF)
