# Supplementary material for: Evolutionary Thinking in Microeconomic Models: Prestige Bias and Market Bubbles
Source: PLoS One. 2013 Mar 27;8(3):e59805. doi: 10.1371/journal.pone.0059805 (PMC3609813; doi:10.1371/journal.pone.0059805)
Supplement: Derivations S1 — This technical appendix reports the equilibria and stability conditions discussed in the main text. Further details can be requested from the author. (PDF) [file pone.0059805.s001.pdf]

Supporting Information:
Evolutionary Thinking in Microeconomic Models: Prestige Bias and Market Bubbles

Adrian V. Bell

Department of Anthropology, University of Utah

December 31, 2012

This technical appendix reports the equilibria and stability conditions discussed in the main text. Further details can be requested from the author.

1 Derivation of price equilibria

The full system of investor behavior and price dynamics derived in the main text is

$$\begin{aligned} x_{t+1} &= x_t + (1 - x_t)p_{(+-)} - (1 + x_t)p_{(-+)} \\ p_{t+1} &= p_t + \beta\left(x_t T_N + T_F(p_{\mathrm{f}} - p_t)\right) \end{aligned} \tag{1}$$

The primary goal is to analyze at equilibrium, given the transition probabilities of becoming an optimistic investor (buyer) ($p_{(+-)}$) or becoming pessimistic (seller) ($p_{(-+)}$).

1.1 Random copying of investor disposition

If we let the transition probabilities equal the current frequency of an investor strategy, i.e.,

$$p_{(+-)} = \frac{1 + x_t}{2},\; p_{(-+)} = \frac{1 - x_t}{2} \tag{2}$$

then it is easy to see that $\Delta x = 0$ and $\hat{p} = p_f + \frac{\hat{x}T_N}{T_F}$, where $\hat{x}$ can be any arbitrary state of optimism among investors.

1.2 Following market trends

Instead of adopting the disposition of random individuals, assume that actors follow market trends such that dispositions change depending on the projected directions of the trade price. An increase in the market value will more likely cause pessimistic speculators to

become optimistic (buyers), and a decrease in market value will cause optimistic investors to more likely become pessimistic (sellers). Then the transition probabilities become:

$$
\begin{aligned}
p_{(+-)} &= \left(\frac{1+x_t}{2}\right)\left(\frac{1}{2} + a(p_{t+1} - p_t)\right) \\
p_{(-+)} &= \left(\frac{1-x_t}{2}\right)\left(\frac{1}{2} + a(p_t - p_{t+1})\right)
\end{aligned}
\tag{3}
$$

There is one equilibrium $(\hat{x},\ \hat{p})$ at $(0,\ p_f)$. At this point the dominant eigenvalue of the linearized system (1) is:

$$
\frac{1 - \beta\left(T_F - 2aT_N\right) + \sqrt{-8a\beta T_N + \left(-1 + \beta(T_F - 2aT_N)\right)^2}}{2}
\tag{4}
$$

after some inspection the eigenvalue is < 1 when

$$
a > \frac{T_F}{2T_N} \quad \text{and} \quad \beta \leq \frac{-2\sqrt{2aT_FT_N} + T_F + 2aT_N}{(T_F - 2aT_N)^2}
\tag{5}
$$

or if,

$$
a < \frac{T_F}{2T_N} \quad \text{and}
\tag{6}
$$

$$
\beta \leq \frac{-2\sqrt{2aT_FT_N} + T_F + 2aT_N}{(T_F - 2aT_N)^2} \quad \text{or} \quad \beta \geq \frac{2\sqrt{2aT_FT_N} + T_F + 2aT_N}{(T_F - 2aT_N)^2}
\tag{7}
$$

1.3 Prestige bias

Described in the main text, the transition probabilities become:

$$
\begin{aligned}
p_{(+-)} &= \left(\frac{1+x_t}{2}\right)\left(\frac{1}{2} + a\left((p_t - p_f) - p_{\mathrm{B}}\right)\right) \\
p_{(-+)} &= \left(\frac{1-x_t}{2}\right)\left(\frac{1}{2} + a\left(p_{\mathrm{B}} - (p_f - p_t)\right)\right)
\end{aligned}
\tag{8}
$$

This specification leads to the investor-price joint equilibria $(\hat{x},\ \hat{p})$ as $(1,\ p_f + T_n/T_f)$, $(-1,\ p_f - T_n/T_f)$, and $(p_{\mathrm{B}}T_n/T_{\mathrm{F}},\ p_f + p_{\mathrm{B}}\ )$. Interested in the stability of the pure optimistic equilibrium ($\hat{x} = 1$, the "growing bubble" condition), we can find the dominant eigenvalue of the linearized system (1) at the equilibrium point $(1,\ p_f + T_n/T_f)$:

$$
1 + 2ap_B - \frac{2aT_N}{T_F}
\tag{9}
$$

which is less than unity when $p_{\mathrm{B}} < T_n/T_f$. Evaluated at the pessimistic equilibrium $(-1,\ p_f - T_n/T_f)$ the dominant eigenvalue is,

$$
1 - 2a\left(p_{\mathrm{B}} + \frac{T_N}{T_F}\right)
\tag{10}
$$

which is always less than unity given $p_{\mathrm{B}} > 0$. Thus the pessimistic equilibrium is always stable, while the optimistic equilibrium is stable when $p_{\mathrm{B}} < T_n/T_f$. Note that unstable internal equilibrium, which determines the domain of attraction for the pessimistic ad optimistic equilibrium, is more in favor of pessimism with a *larger* fraction of the trading coming from non-fundamentalists.
